# Hybrid nickel-free graphene/porphyrin rings for photodegradation of emerging pollutants in water†

**DOI:** 10.1039/c9ra06328e

**Published:** 2019-09-24

**Authors:** Martina Ussia, Mario Urso, Maria Miritello, Elena Bruno, Giusy Curcuruto, Daniele Vitalini, Guglielmo G. Condorelli, Maria Cantarella, Vittorio Privitera, Sabrina C. Carroccio

**Affiliations:** University of Catania, Department of Physics and Astronomy “Ettore Majorana” Via Santa Sofia 64 95123 Catania Italy; CNR-IMM Catania Via Santa Sofia 64 95123, Catania Italy sabrinacarola.carroccio@cnr.it; CNR-IPCB Via Paolo Gaifami 18 95126, Catania Italy; University of Catania, Department of Chemistry and INSTM UdR Catania Viale Andrea Doria 6 95125 Catania Italy

## Abstract

A novel hybrid photoactive material based on graphene foam (G) coupled with porphyrin-based polymers (Porph rings) was formulated by using a time-saving procedure to remove nickel from the final device. Specifically, Porph rings were spin coated onto the G platform with the double function of a visible-light photocatalyst and protective agent during nickel etching. The characterization of G-Porph rings was assessed by Scanning Electron microscopy (SEM), X-ray photoelectron spectroscopy (XPS) and photoluminescence (PL). The novel material showed photocatalytic ability in degrading different classes of pollutants such as the herbicide 2,4 dichlorophenoxyacetic acid (2,4-D), polyethylene glycol (PEG) as an ingredient of care and health products, and also the methylene blue (MB) dye. UV-Vis spectroscopy, total organic carbon (TOC) and soft mass spectrometry techniques were used to monitor the photocatalytic process. The best performance in terms of photocatalytic efficiency was exhibited *versus* PEG and MB degradation. Furthermore, to determine the individual contribution of Reactive Oxygen Species (ROS) produced, free radical and hole scavenging tests were also carried out. Finally, a detailed map of the photocatalytic degradation mechanisms was proposed, reporting also the calculation of Porph rings' Highest Occupied Molecular Orbital (HOMO) and Lowest Occupied Molecular Orbital (LUMO) energy level values.

## Abstract 2

A novel hybrid photoactive material based on graphene foam (G) coupled with porphyrin-based polymers (Porph rings) was formulated by using a time-saving procedure to remove nickel from the final device.

## Graphical abstract

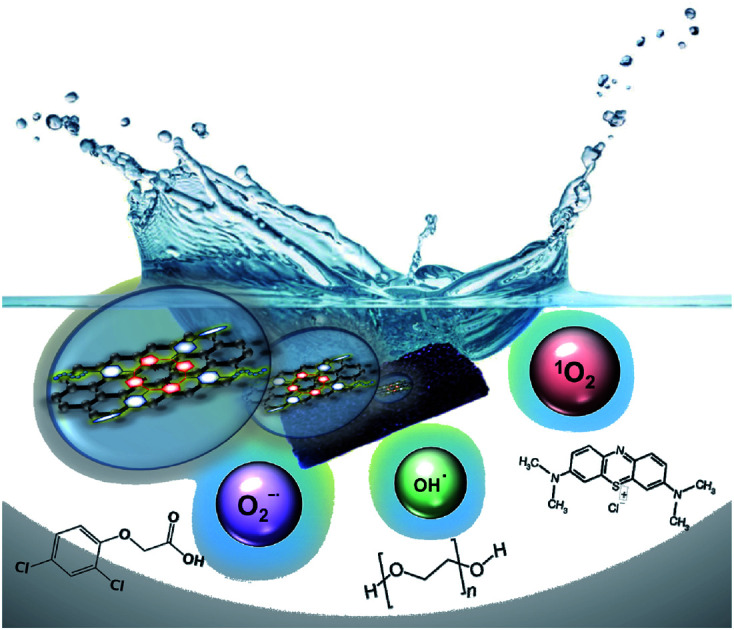

## 1. Introduction

The light induced processes enacted by organic semiconductors represent a promising environmentally friendly solution in several fields such as medicine, energy and water treatment applications.^1–3^ In particular, wastewater treatment driven by photocatalysis takes advantage of the use of light as a renewable energy source, allowing purification without adding further chemicals to the aqueous media.^4^ Importantly, photocatalytic based technologies can lead to the removal of emerging contaminants such as drugs, pesticides and chemicals deriving from personal care products (PCPs), becoming a more and more appealing approach to improving the traditional purification systems.^4–8^ The efficiency of this technology is based on the production of reactive oxygen species (ROS) in water, which are able to mineralize almost all organic pollutants. As well stated in the literature,^9^ visible-light photocatalysis is mainly triggered by photosensitizers (*i.e.* porphyrin, phtalocianine…) through a spin forbidden intersystem crossing (ISC) process, leading to the production of singlet oxygen (^1^O_2_), which is the most reactive ROS specie.^10,11^ In this context, porphyrins represent one of the most used class of light-induced catalysts, finding applications in many fields including water remediation.^9–16^ Nevertheless, the ISC for such kind of materials is strongly limited by rapid electron–hole recombination between the singlet excited state and the ground state, mainly due to aggregation phenomena.^13,16^ To turn this limitation, the combination of porphyrins with carbon-based nanostructures, such as carbon nanotubes and graphene^17–23^ was exploited. Indeed, these materials are considered ideal candidates as co-catalyst carriers, having specific features such as high-surface area and conjugated networks.^20,21^

It was demonstrated as the non-covalent assembly of porphyrin based polymers (Porph rings) with a graphene platform supported by nickel, represents an efficient method to avoid the electron–hole recombination mechanism as well as other critical issues deriving from the use of G derivatives.^19,24–26^ The present paper describes the formulation of a novel nickel free photoactive material, its characterization as well as the related photocatalytic attitudes on different classes of contaminants. Undeniably, Ni removal is strictly required for applications in water and medical fields, being an allergen and a water contaminant (≥0.010 ppm) harmful for aquatic and human life.

In light of this, a time-saving method to remove Ni from G avoiding the collapse of the 3D structure during the nickel-etching was developed.^27^

3D collapse constitutes critical issue for the production of G foam from a Ni template by chemical vapor deposition (CVD).^28^ After growing onto the Ni substrate, the production of G foam required a multi-step process which includes polymer infiltration, etching by acidic solution, solvent washing to dissolve the polymer coating and finally, drying or lyophilization. These latter two steps are crucial to obtain a foam-like network graphene, avoiding its structural collapse.

The photocatalytic activity of as prepared hybrid material was monitored by UV-Vis, TOC and MS techniques selecting as target pollutants the pesticide 2,4-dichlorophenoxyacetic acid (2,4-D), MB dye, and the water soluble polyethylene-glycol (PEG). The 2,4-D herbicide is widely used in agriculture, although its toxicity is well known along with a poorly water biodegradability.^29,30^ Concerning the PEGs, controversial opinions were reported on its safe environmental use. This hydrophilic polymer, which is often included in several copolymeric formulations for a wide range of applications, is considered biodegradable up to 10 monomeric units. However higher molar masses of PEG are commonly used for cosmetics, detergents and biomedical purposes, becoming an insidious threat for the environment.^31–34^ Finally, MB being one of the most used simulant dye, was selected as a reference to compare our results with data reported in the literature,^22,35–37^ as well as to determine and discriminate among the ROS production during photo-process.

## 2. Results and discussion

The graphene platform used to prepare the device was obtained by CVD deposition on a Nickel foam template (see experimental section). To cover the nickel–graphene foam (Ni–G), a water insoluble polymeric materials based on porphyrin rings having a *M*_w_ of 10 600 Da were properly selected^19,38^ as photoactive coating film. The hybrid material preparation is reported and discussed in the following sections.

### 2.1 Nickel free–graphene foam/Porph rings (Ni free–G/Porph rings) composite preparation

**Fig. 1:**
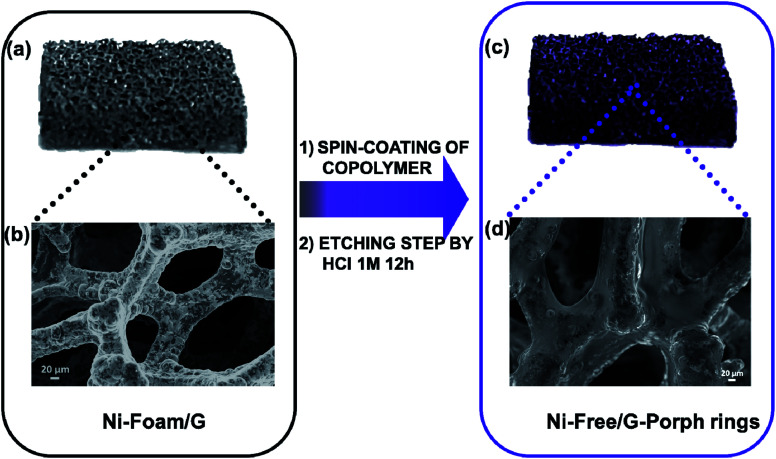
(a) and (b) As-grown 3DG coated with a thin layer of Porph rings by a spin-coating deposition method and (c) and (d) Ni free-G/Porph rings sample after etching by hot HCl 1 M solution maintaining the typical 3D graphene structure.

As well stated, the etching procedure to eliminate the nickel catalyst substrate requires the graphene protection by spin-coating polymers such as commercial poly-methylmethacrylate (PMMA) or polydimethyl siloxane (PDMS).^39^ Subsequently, the sample is etched using HCl 3 M, dried and immersed in hot-solvent to remove the polymer, obtaining the final product to employ. However, this step induces a 3D structure collapse caused by the liquid capillary force involved during solvent evaporation, that provides a thinner G than the pristine Ni-Foam.^40^ Thus, polymer removal drastically limits the use of Ni free–G as freestanding materials. In recent papers, concerning the formulation of conducting G foam devices, the collapse of the 3D structure was avoided by non-removing the protective polymer coating that remains as a part of the final material.^39,41^ Taking into account this strategy, the novelty here reported consists in preparing the Ni free sample in a one-step process preserving *via* photocatalyst coating, the G support [see Fig. 1a–d]. In particular, Porph rings were used with the double function of the photoactive part of the device and protective coverage, as well. To successfully deposit the polymer by spin-coating, Porph rings were synthesized with an appropriate *M*_w_ and characterized by using gel permeation chromatography and mass spectrometry (Table S1† and Fig. S1a and b†). Thus, the hybrid system was etched. This treatment avoided the collapse of the network obtaining the final photoactive material without any additional step. The as-prepared nickel free sample was subjected to morphological and physico-chemical characterizations reported as follows.

### 2.2 Graphene 3D characterization

#### 2.2.1 SEM analysis

**Fig. 2:**
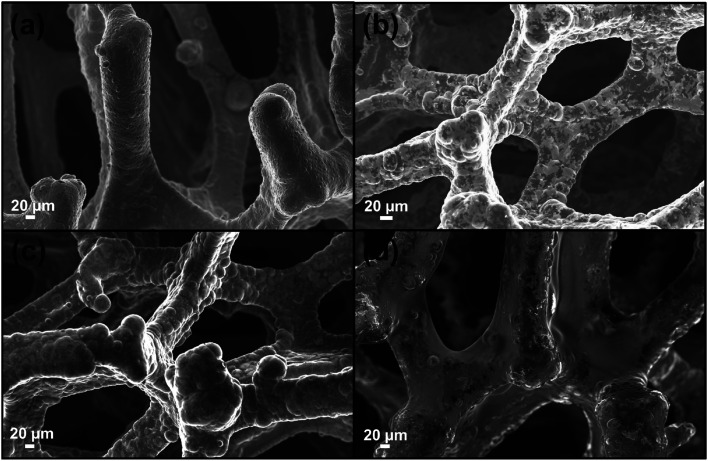
SEM images obtained in the inlens mode of (a) Ni-foam (b) Ni-Foam/G (c) Ni-Foam/G-Porph rings (d) Ni-free/G-Porph rings.

The pristine Ni-foam possesses a 3D porous structure and a tortoiseshell-like morphology [see Fig. 2a] with pores sizes ranging from 60 to 130 μm. The SEM images of the as-deposited graphene are reported in Fig. 2b revealing that the graphene adheres to the surface of the nickel foam, reproducing its morphology.^19,38^ As described in the experimental part, the G was drop-coated with the cyclic polymer obtaining a homogeneous coverage, as shown in Fig. 2c. Fig. 2d shows that the morphology of the Ni-free/G-Porph rings sample appears unchanged after the etching procedure, demonstrating the efficacy of the polymer-layer onto the graphene surface to protect the G network from the collapse of the structure.

#### 2.2.2 XPS analysis

**Fig. 3:**
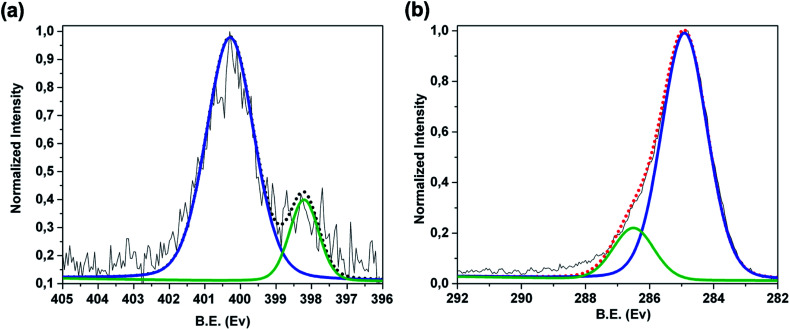
XPS spectra of Ni-free/G-Porph rings sample in the (a) high resolution N 1s region and (b) high resolution C 1s region.

To ascertain that the etching procedure does not affect the nature of the polymer coating and its related interactions with the G substrate, we performed XPS analysis. Furthermore, XPS provides also information about the effective removal of Nickel. Fig. 3a reports the XPS N 1s region of the Ni-free/G-Porph rings sample. The signal consists of two components centered at 400.2 eV and at 398.2 eV, indicating the presence of the pyrrolic (–NH–) and to the iminic (–N=) species of the porphyrin rings respectively.^19,41,42^ Furthermore, the C 1s region [Fig. 3b] reveals two components positioned at 285.0 eV (FWHM of 1.5 eV) and at 286.5 eV (FWHM of 1.7 eV), due respectively to the C–C, C=C, C–H of the graphene and to the C–O, C=N, C–N species of the 3DG copolymer-porphyrin structure.

Fig. S2 (see ESI†), reporting the Ni 2p region, does not show the typical signals of Ni suggesting that the metal was effectively removed. However, to confirm the nickel removal after etching, we also performed a quantitative analysis by using Inductively Coupled Plasma-Mass Spectrometry (ICP-MS). In Table 1 the nickel contents are reported before and after sample etching. As it is possible to observe, the residual Ni amount after the etching procedure, was lower than 100 μg cm^−2^. Furthermore, during the photocatalytic treatment, after 6 hours of irradiation, the Ni content in water was measured, obtaining a value of 20 μg cm^−2^.

**Table 1:** Nickel content in G samples before and after etching

Ni-Foam/3DG	Ni-Foam/Porph rings	Ni-Free/3DG Porph rings	Ni-Free/3DG Porph rings (after 6 h irradiation)
165 736 mg cm^−2^	166 066 mg cm^−2^	94 μg cm^−2^	20 μg cm^−2^

#### 2.2.3 Photoluminescence measurements

**Fig. 4:**
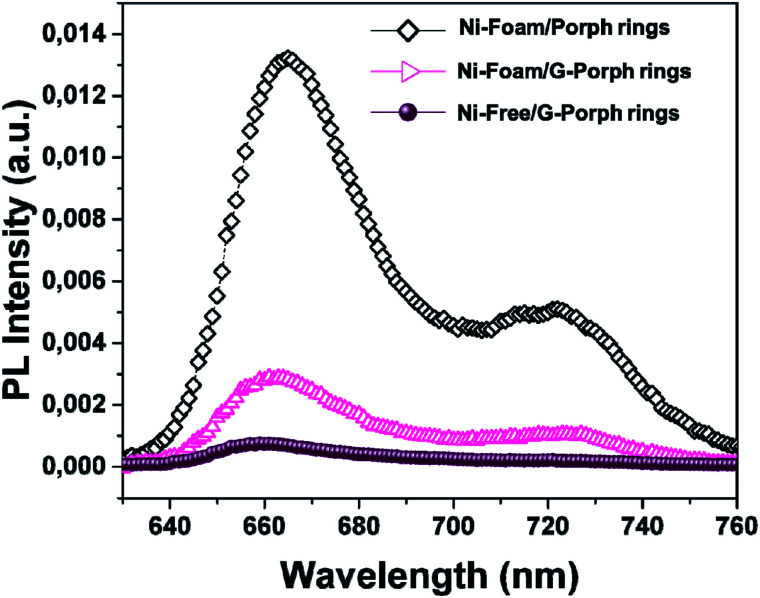
PL spectra of Ni-Foam/Porph rings (black rumble), Ni-Foam/G-Porph rings (fuchsia triangle), Ni-free/G-Porph rings (purple circle) collected upon excitation at 325 nm.

The charge electron–transfer processes in hybrid systems were evaluated by analyzing the influence of the G presence on the optical emission of the polyporphyrins.^43,44^ Since PL emission strongly depends on the radiative recombination rate of excited electron–hole pairs, additional electron pathways occurred. As reported in the literature, coupling of photoactive systems with graphene-based materials produces a quenching in the PL intensity induced by additional electron-transfer process due to the G interaction at material interfaces.^22,23^Fig. 4 compares the PL emission collected under excitation at 325 nm on Ni-foam/3DG and on Ni-free/3DG with a reference Porph rings copolyformal on pristine Ni-foam (Ni-Foam/Porph rings). In all cases the PL emission exhibits a broad band composed by two peaks centered at 660 and 720 nm, related to their *Q*_*X*_ (0–0) and *Q*_*X*_ (0–1) transitions respectively.

However, an effective quenching in the case of Ni-foam/G-Porph rings and a quasi-total disappearance of characteristic emission band for the Ni-Free/G-Porph rings, with a slightly blue-shift of the signals are evident. This key result validates that π–π interactions between porphyrin aromatic moieties and graphene take place, allowing a remarkable electron transfer at the polymer interface.^44,45^ Different phenomena could justify the further PL quenching after nickel removal, such as the change in graphene electronic structure as well as an enhanced graphene π-delocalization after electron injection.^20,43,45,46^

#### 2.2.4 Cyclic voltammetry measurements

**Fig. 5:**
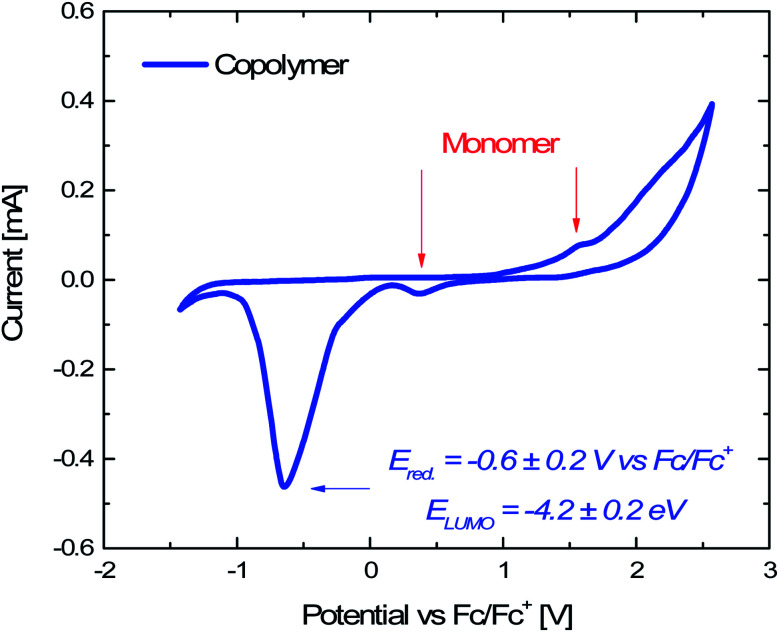
Cyclic voltammetry of Porph rings recorded at 25 mV s^−1^ scan rate in 0.1 M TBAPF6 in dichloromethane solution. Ferrocene was used as internal standard.

To gain a better insight into the photodegradation mechanisms, it is very important to understand the electronic properties of the Porph rings (*i.e.* HOMO and LUMO levels). Although the cyclic voltammetry (CV) represents an appropriate tool to estimate the Frontier orbitals, it often requires the combination with other techniques, such as UV-Vis spectroscopy.^47,48^ In this work, CV curves were referenced to ferrocene by taking *E*_1/2_(Fc/Fc^+^) = 0.429 V *vs.* SCE (Fig. S3†). From the inspection of the CV measurements on the Porph rings reported in Fig. 5, the small oxidation and reduction peaks at ∼1.6 and ∼0.4 V *vs.* Fc/Fc^+^ are attributed to traces of the residue monomer molecules, as demonstrated by the CV curve of the porphyrin monomer solution (Fig. S5†). The presence of residue porphyrin monomer was also confirmed by the MALDI spectra (Fig. S1†). Thus, the high and irreversible reduction peak at −0.6 ± 0.2 V *vs.* Fc/Fc^+^ was ascribed to the Porph rings (half of FWHM was used to evaluate the error). HOMO (*E*_HOMO_) and LUMO (*E*_LUMO_) energy levels can be estimated from oxidation (*E*_ox_) and reduction (*E*_red_) peaks potential as follows:*E*_HOMO_ = −(*E*_ox_*vs.* Fc/Fc^+^ + 4.8) eV 1*E*_LUMO_ = −(E_red_*vs.* Fc/Fc^+^ + 4.8) eV 2where 4.8 eV is the formal potential of the Fc/Fc^+^ redox couple in the Fermi scale.^48^ CV curves were referenced to ferrocene by taking *E*_1/2_(Fc/Fc^+^) = 0.429 V *vs.* SCE (Fig. S5†). According to eqn (2), the LUMO energy level of the Porph rings was estimated to *E*_LUMO_ = −4.2 ± 0.2 eV. On the other hand, by UV-Vis absorption measurement (Fig. S6†) we measured the optical bandgap (*E*_g_) as *E*_g_ = 2.2 eV and thus we estimated the HOMO energy level as *E*_HOMO_ = *E*_LUMO_ − *E*_g_, finding the eqn (1)*E*_HOMO_ = −6.4 ± 0.2 eV.^49–51^ To our knowledge, this is the first time that HOMO and LUMO energy levels of cyclic porphyrins-based copolyformal are measured by CV.

## 3. Photocatalytic degradation tests

**Fig. 6:**
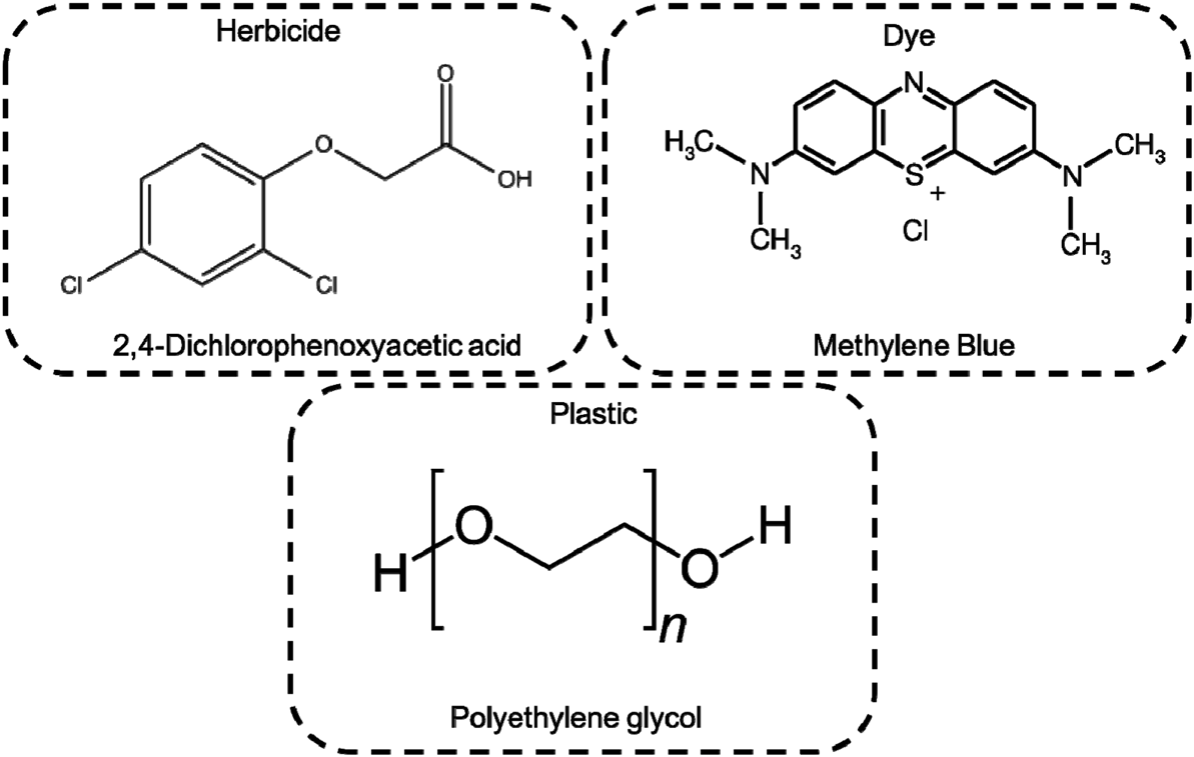
Structures of tested pollutants.

The photocatalytic activity of Ni-free/G-Porph rings under Visible-Light (VL) irradiation was tested by monitoring the degradation of MB, 2,4-D and PEG (see Fig. 6).

**Fig. 7:**
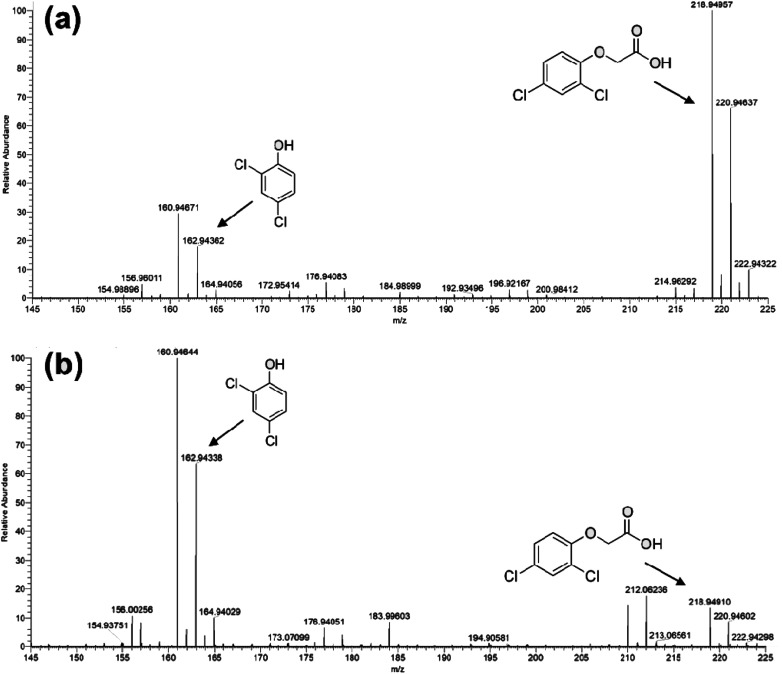
ESI-MS spectra collected in negative ion mode for: (a) untreated 2,4 D solution and (b) Visible-light photo-exposed 2,4-D solution after 6 hours of irradiation time.

In Fig. 7a–b the ESI-MS spectra of (a) 2,4-D pristine solution and (b) after 6 hours of visible-light irradiation in the presence of the photocatalyst are reported. As shown from the spectra, the ESI-MS peaks associated to the 2,4-D molecules (*m*/*z* at 218.95/220.95), disappeared after the photocatalytic procedure. Conversely, the lesser abundant signals at *m*/*z* 160.95/162.94 assigned to the main degradation product 2,4-dichlorophenol,^30^ significantly increased after the photocatalyst addition. The initial presence of 2,4-dichlorophenol molecules in the pristine sample derived from synthesis impurity. Although the formation of by-products indicated the occurrence of herbicide degradation, 2,4-D mineralization was not successfully achieved under these experimental conditions. Indeed, as revealed from UV-Vis and TOC analyses (see Fig. S7†), absorbance profiles as well as TOC values remained unchanged during the photo-exposure, most likely due to the formation of recalcitrant 2,4-dichlorophenol and other chlorinated species in solution. As expected, these intermediates are very difficult to mineralize by vis-light photocatalytic process^52,53^ requiring more severe experimental conditions.

**Fig. 8:**
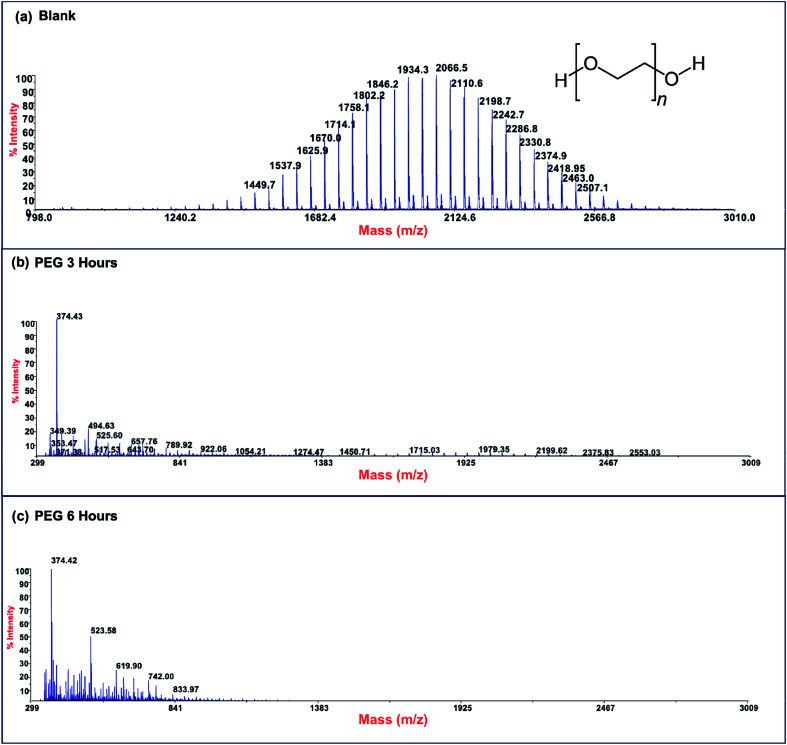
MALDI-TOF spectra of (a) pristine PEG sample and after (b) 3 hours and (c) 6 hours of photo-exposure.

**Fig. 9:**
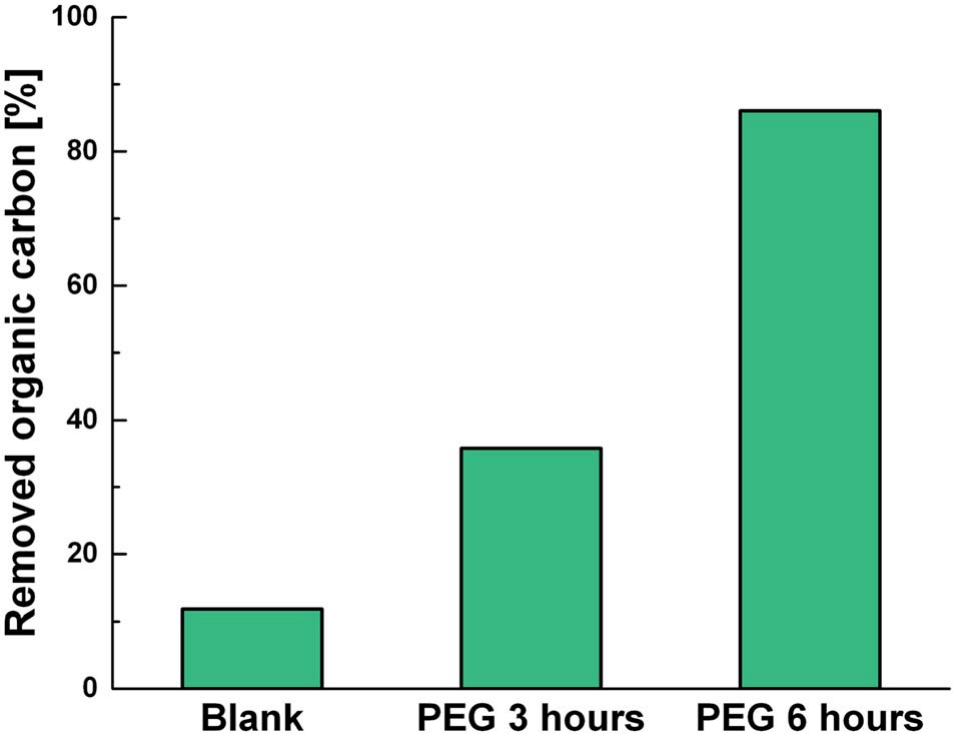
TOC measurements of pristine PEG irradiated for 6 hours (Blank) and PEG treated with Ni-free/G-Porph rings after 3 hours (PEG 3 hours) and 6 hours (PEG 3 hours) of photoexposure.

Photodegradation ability of hybrid material was tested also on PEG solution. Among water soluble polymers, PEG is constituted by a simple molecular structure representing an adequate compound for studying photocatalytic degradation features. To this purpose, a sample having a narrow polydispersity (*D* = 109) with an *M*_w_ of 1900 Da was selected to allow the simultaneously determination of molar masses and degradation products as well, by MALDI methods during photoexposure. Fig. 8a–c reported MALDI TOF spectra collected in reflectron mode of (a) pristine PEG sample; after (b) 3 hours and, (c) 6 hours of photo-exposure in the presence of the photocatalyst. As it is possible to notice from the figure, relative abundance of signals corresponding to PEG macromolecular chains, significantly decreased (see also Fig. S8†) as the exposure time proceeded, producing oxidized polymer chains at lower mass range. Degradation products deriving from the photocatalytic process are assigned to PEG oligomers having formic acid, aldehydic and alcoholic end groups (see Fig. S9†). After 6 hours, progressively degradation took place, leading to the disappearing of initial PEO distribution with the formation of much lower macromolecular oxidized chains, that cannot be detected being lower than the mass range inferior limit. Photochemical oxidation mechanism proposed in the literature for PEG^32–34,51^ well fitted with the products revealed by MALDI, involving mainly the ROS attack on CH_2_ groups with a random chain scission pathway. At this stage mineralization measured by TOC revealed 85% of PEG removal (Fig. 9).

**Fig. 10:**
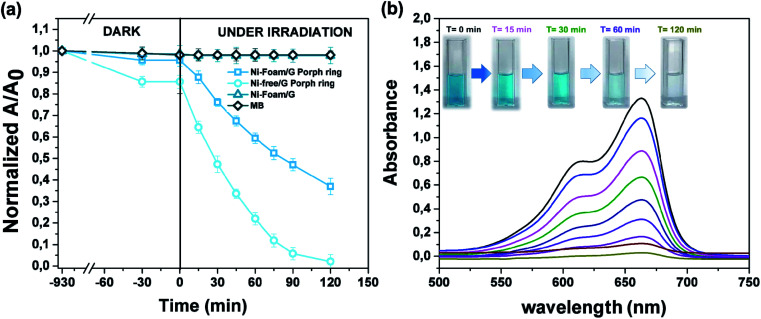
(a) Photocatalytic activity of Ni-Foam/G, Ni-Foam/G- Porph rings, Ni-Free/G-Porph rings toward the degradation of MB after 120 minutes of light irradiation; (b) time dependent absorption changes of MB upon visible irradiation in the presence of Ni-free/G-Porph ring.

The photocatalytic activity *versus* MB (Fig. 10) was also tested, by comparing the results with those reported in the literature for similar devices where dyes were used as reference pollutants.^54,55^ The dark adsorption data of all the samples are reported in Fig. 10a evidencing a major adsorption in the case of the Ni-free material. This value was most likely due to hollow channels of G, formed after Ni removal and exposed to the MB solution. When the adsorption/desorption equilibrium was reached, samples were exposed to the visible light.

Data collected after 120 minutes of light irradiation revealed that Ni-free/G-Porph rings degraded 93.4% of MB [see also Fig. 10b], whereas the Ni-foam/G-Porph rings showed 60% of photo-degradative capacity. As expected, the Ni Foam/G, used as external reference did not exhibit any MB degradation. These results indicate that the Ni-removal plays a key-role in the photodegradation, eliminating almost all the MB after only 120 minutes of VL irradiation. According with PL measurements, the boost of the photocatalytic efficiency was reasonable due to a more effective electron-transfer process at the graphene interface. Compared with the performance of similar devices^54,55^ monitored by using dyes degradation tests, this hybrid sample offered the great advantage to use a standalone system,^19,54^ containing lower amount of photocatalysts (0.4 mg cm^−2^),^55,56^ to obtain valuable photocatalytic properties under visible light irradiation.

### 3.1 ROS determination

The photochemistry of the material was investigated by the qualitative evaluation of the ROS produced, by following methods described in ref. 21, 32 and 33. Generally, when a photocatalyst is irradiated by light, ROS such as hydroxyl radical (OH˙), superoxide radicals (O_2_˙^−^) and the singlet oxygen (^1^O_2_) are generated.^54^ These reactive species are able to degrade the pollutants by redox reactions.^9^ If compared with inorganic photo-catalysts, the formation of ^1^O_2_ is a distinctive feature of photosensitizers. The singlet oxygen is a selective oxidant specie able to promptly react with unsaturated double bonds, phenols, sulfides, amines and other electron-donor compounds.^57^ In addition, it explicates a highly cytotoxic action *versus* cells and microorganisms becoming particularly attractive in wastewater treatment for its dual function of decontamination and disinfection.^58^

**Fig. 11:**
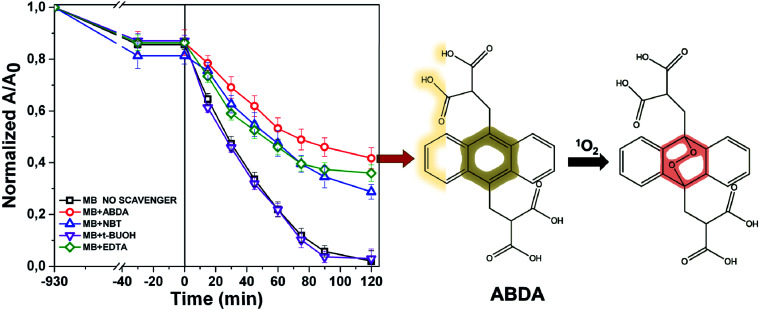
Effect of ABDA, NBT, EDTA and *t*-BuOH radical-hole scavengers on the degradation rate of MB by using the Ni-free/G-Porph rings sample. The inhibition of the MB degradation due to the ABDA oxidation indicates the effective ROS production during 120 minutes of irradiation time. Among the produced ROS, ^1^O_2_ constitutes the predominant species.

**Fig. 12:**
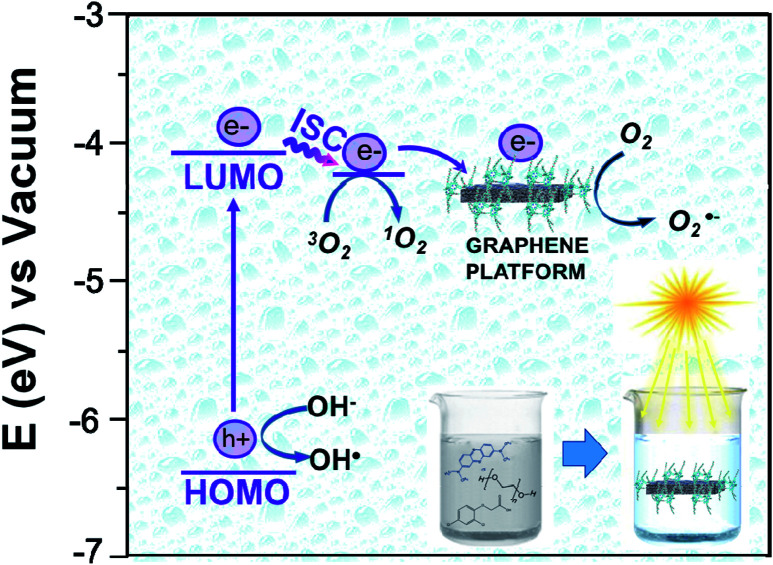
Energy diagram of the proposed mechanism for the photocatalytic degradation of MB by using Ni-free/G-Porph rings as organic photosensitizer.

The discrimination among the produced ROS can be obtained by introducing specific radical-trapping agents (Fig. 11) during the light exposure. These scavenger molecules interacting with the related photo-generated radicals, inhibit their reaction with the organic pollutant. In particular, ethylene diamine tetraacetic acid (EDTA), *t*-BuOH, nitro-blue tetrazolium (NBT) and 9,10-anthracene-diyl-bis(methylene)dimalonic acid (ABDA), can be efficiently used to scavenge photo-generated holes, hydroxyl radical, superoxide radical and singlet oxygen respectively.^35,36,57^ Consequently, the photochemistry mechanisms involved during photoexcitation can be better elucidated. In our experiments, samples of 1 cm^2^ of Ni-free photocatalyst are immersed in MB solutions up to the adsorption–desorption equilibrium. Then, increasing volumes of trapping agents from 1 mg L^−1^ up to 10 and 15 mg L^−1^ are added to the solutions evaluating their inhibitory effect on MB degradation (Fig. 11 and S10–S13†). In Fig. 11 the degradation tests collected by adding 15 ppm of trapping agents, together with the MB degradation trace of our sample are displayed, and compared. As it is possible to observe from Fig. 11, the addition of *t*-BuOH did not affect MB photo-degradation trend. On the contrary, when EDTA is added to the solution, the MB experienced only a decrement of 52% denoting as photo-generated holes took part to the process. Similarly, the presence of NBT and ABDA seem to determine inhibition of MB degradation. As well known, their scavenger effects can be referable to the presence of superoxide radical and singlet oxygen respectively. These findings are entirely in agreement with recent literature reporting similar systems, proposing also an increase of singlet oxygen quantum yield induced by graphene–porphyrin π–π interactions.^23,54^ On the basis of these experimental data, MB degradation mechanisms of the Ni-free/G-Porph rings are here proposed in Fig. 12.

The work function (*E*) of graphene *versus* vacuum can be calculated to be about 4.42 eV.^34^ Considering the previous reported electrochemical measurements, we can also assign and plot the LUMO (−4.2 eV) and HOMO (−6.64 eV) energy levels of our sample. Based on these data, the light-excited porphyrin electrons can be excited from its S_0_ to the 
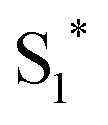
 LUMO level. Here, the excited state 
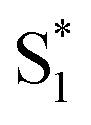
 can involve ISC resulting in a first excited tripled state 
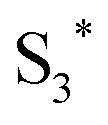
 as shown in eqn (1). Supported by the literature, we can reasonable suppose that this process is also facilitated by the presence of graphene, which can prevent the 
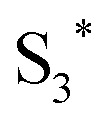
 physical deactivation^48^ and the electron–hole recombination between 
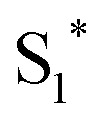
 and the porphyrin ground state.^54,57^ Since 
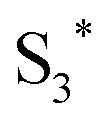
 has a longer lifetime than 
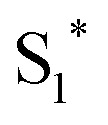
,^7^ it can transfer its energy to another molecule. In the presence of oxygen, 
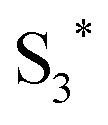
 transfers its energy to the oxygen triplet ground state, producing singlet oxygen (2).1
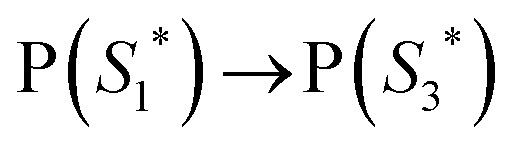
2
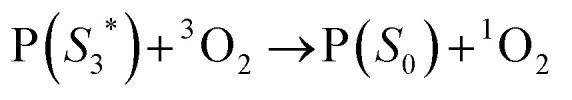


At the same time, 
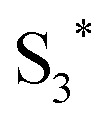
 can transfer electrons to oxygen (3) as well as graphene, generating superoxide radicals (O_2_˙^−^), in accordance with our radical-trapping experiments.3
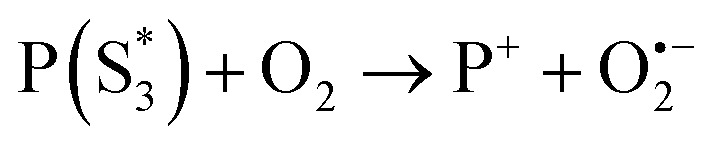


## 4. Conclusions

The hybrid Ni-free/graphene-Porph rings (Ni-free/G-Porph rings) was here proposed as a new photocatalytic system. The spin-coated photocatalyst was able to protect G during the Ni etching from defects formation and collapse as well. At the same time, the desired π–π interactions at the G/polymer interface, crucial to promote the electron transfer process were strongly achieved as confirmed by PL and photocatalytic degradation measurements. The freestanding material obtained after Ni removal, exhibited diverse photocatalytic performance depending on the selected target pollutant. Specifically, the Ni free device was able to degrade 2,4-D herbicide, without reaching mineralization. Differently, under the experimental condition used, the PEG sample was photodegraded and mineralized according to TOC results. The radical trapping tests performed by MB solution, confirmed that singlet oxygen is the predominant species, although the other ROS are also produced assisting the contaminants degradation. Finally, a detailed map of photo-mechanisms was reported, including the calculation of the Highest Occupied Molecular Orbital (HOMO) and Lowest Occupied Molecular Orbital (LUMO) energy level values of the photosensitizer, calculated by the electrochemical study.

## 5. Experimental

### 5.1 Materials and reagents

All solvents and basic materials were commercial products appropriately purified before use.

All chemicals were used as received, unless otherwise noted. 5,10-di[*p*-(9-methoxytriethylenenoxy)phenyl]-15,20-di[*p*-hydroxyphenyl]-porphyrin and 1,20-di(bisphenoxy-A) eicosane monomer were synthesized and characterized according to our previous report^19^ (Table S1,† Fig. S1†). Nickel foam is purchased from GoodFellow with following features: 0.45 g cm^−2^, porosity 95%, 20 pores per cm, purity 95% and 1.6 mm thickness.

### 5.2 Molar masses determination

Size Exclusion Chromatography (SEC) analyses were performed by using a Waters 515 HPLC pump, having four Ultra-Styragel HR columns in series (in the order: HR4, HR3, HR2 and HR1), and a Waters R401 differential refractometer. 1 mg ml^−1^ of THF solutions, were introduced at a flow rate of 1 mL min^−1^. Calibration points were acquired by using selected polymethylmethacrylate references.

### 5.3 Electrospray ionization mass spectrometry

ESI mass analysis was performed by an Orbitrap Exactive mass spectrometry equipped by a H-ESI source. Mass spectra were acquired in negative ion mode in the *m*/*z* range 150–225 under the following conditions: capillary temperature 130 °C, nebulizer gas (nitrogen) with a flow rate of 15 arbitrary units; source voltage 4 kV; capillary voltage 46 V; tube lens voltage 90 V. After photodegradation process, each samples was injected (direct infusion) at a flow rate of 10 μL min^−1^. The mass spectrometer was calibrated using a standard mixture sodium dodecyl sulfate (265.17 Da), Ultramark (1621 Da) and sodium taurocholate (51 442 Da).

### 5.4 Matrix assisted laser desorption ionization-time of flight (MALDI-TOF)

Spectra were acquired in reflector positive-ion mode using a 4800 MALDI TOF/TOF™ Analyzer, supplied with a Nd : YAG laser. Mass calibration was obtained by means of a set of polypeptides having different molar masses. Sample preparation was achieved by mixing 0.1 mmol of analytes and 40 mmol of *trans*-3-indoleacrylic acid for porphyrin polymers sample and *trans*-2-[3-(4-*tert*-butylphenyl)-2-2-methyl-2-propenylidene]malononitrile for PEG, using THF as a solvent for both polymers.

### 5.5 Determination of total organic carbon (TOC)

The mineralization of the pollutants after irradiation in the presence of samples was evaluated by measuring the TOC content with a TOC analyzer (Shimadzu TOC-LCSH) equipped with a non-dispersive infrared detector.

### 5.6 Inductively coupled plasma–mass spectrometry (ICP-MS)

ICP-MS analysis was performed by using a NEXION 300X (PerkinElmer). A piece of 1 cm^2^ of Ni-free/3DG copolymer was mineralized (700 W, 40 minutes). 10 ppb of internal standard (Multielement standard solution for ICP-TraceCERT®) was added.

### 5.7 X-ray photoelectron spectroscopy (XPS) analysis

Multi technique spectrometer PHI ESCA/SAM 5600 equipped with Mg standard X-ray source was used to obtain XPS data. The measurements were setted at 45° photoelectron take-off angle in relation to the target sample with an angle of ±3°. was set at 23.5 eV for high-resolution spectra were collected by fixing at 23.5 eV. The analyzer pass energy. Calibration of the binding energy (BE) was achieved by centering the C 1s peak at 285.0 eV. The analyses were executed on 2 mm diameter surface area of the sample.

### 5.8 Scanning electron microscopy (SEM) analysis

Hybrid structure morphologies were obtained by scanning electron microscopy (SEM, Gemini 152 field emission SEM Supra 25, Carl Zeiss, Germany) working at 2.0 kV, in the inlens operation mode.

### 5.9 Photoluminescence (PL) analyses

PL measurements were performed by sampling the same surface areas of the photocatalyst and reference as well (1 cm^2^), at room temperature by an He–Cd laser (excitation wavelength of 325 nm). PL signals have been recorded by a monochromator and a water-cooled Hamamatsu photomultiplier.

### 5.10 Synthesis of nickel/graphene foam (Ni/G)

Synthesis of Ni-G samples was performed in AIXTRON's Black Magic Chemical Vapor Deposition (CVD) system.^19^ Systems were heated at 900 °C for 20 minutes under Ar and H_2_ flows (600 and 400 sccm, respectively) to eliminate the residual thin surface oxide layer before graphene growth. After, the chamber was heated up to 1000 °C and the CH_4_ flow was opened for 40 minutes (flow rates of 20, 600 and 1000 sccm for CH_4_, Ar and H_2_, respectively) and then cooled down at 15°C min^−1^ to grow the graphene layers on the Ni foam surface.

Characterization of graphene in terms of quality and number of layers was performed by Raman spectra.^19^

### 5.11 Ni free-G/porph rings preparation

About 10 mg of Porph rings was dissolved in 1 mL of dimethylformamide (DMF). G pieces of 1 cm^2^ were covered by spin-coating a DMF solution 10 mg mL^−1^ of porph rings. The specimens were dried on a hot plate (80 °C) for 40 minutes and overnight in a under vacuum oven at 60 °C. Then, samples were immersed in HCl (1 M) for 12 hours. Finally, after washing in fresh distilled water the samples were dried in oven for two hours at 45 °C.

### 5.12 Cyclic voltammetry measurements

Electrochemical analyses were performed to measure the HOMO and LUMO energy values of the Porph rings by using a potentiostat (VersaSTAT 4, Princeton Applied Research, USA) and a three-electrode setup with Pt wires as working and counter electrodes, a saturated calomel electrode (SCE) as reference electrode and 0.1 M tetrabutylammoniumhexafluorophosphate (TBAPF6) as supporting electrolyte. The experiments were carried out on three different dichloromethane solutions containing 1 mg of porphyrin copolymer, 1 mg of porphyrin monomer (Fig. S3†) and TBAPF6 (Fig. S4†). For each experiment dichloromethane solvent was preventively passed in molecular sieves and ferrocene was used as an internal standard (Fig. S5†). To evaluate the HOMO energy level we measured the optical bandgap (*E*_g_) from the maximum value of the *Q*_*y*_ (0,0) absorption band (554 nm) of UV-Vis spectrum of Porph rings (Fig. S6†) by using *E*_g_ = *hc*/*λ*_onset_ where *h* is the Planck constant (6.63 × 10^−34^ m^2^ kg s^−1^) and *c* is the speed of light (3 × 10^8^ m s^−1^).

### 5.13 Photocatalytic tests

The photocatalytic efficiency of the hybrid material was evaluated *versus* 2,4 D, PEG having a *M*_n_ 1900 Da, as well as MB water solutions under visible-light irradiation. All solutions were saturated by bubbling air every 10 minutes per hours, and were irradiated by the solar simulator VeraSol-2 from Oriel by Newport corresponding to AM1.5 G in the visible and near-infrared region (400–1100 nm) with a light irradiance of 100 ± 3 mW cm^−2^. Specifically, MB degradation tests were performed by removing the irradiation in the range 600–700 nm to suppress MB absorption (see Fig. S14†). A 1 cm^2^ sample was immersed in 2 mL of the target solution (0.015 Mm) and left overnight in the dark up to the adsorption–desorption equilibrium. The variation of MB concentrations was spectrophotometrically evaluated observing the absorption at 664 nm. The photodegradation ratio was defined as (*A*_0_ − A)/*A*_0_ where A is the absorption of the dye at a certain time and *A*_0_ represents the absorption value of the MB initial concentration.

With regards to 2,4-D photodegradation tests, 1 cm^2^ of device was immersed in 2 mL of herbicide solution (0.005 mM) and irradiated for 6 hours. Control experiments were carried out in the dark for 30 minutes to ensure the adsorption–desorption equilibrium of the herbicide. The photodegradation products were determined by the ESI-MS as well as UV-Vis measurements, following also the degradation by UV-Vis and TOC analyses.

Finally, to evaluate the photodegradation ability *versus* polyethylene-oxide (PEG), 1 cm^2^ of speciment was immersed in 2 mL of polymer aqueous solution (0.5 mg mL^−1^). To estimate the photodegradation process, MALDI-TOF spectra were acquired up to 6 hours of photoexposure, while mineralization of PEG was monitored by measuring the TOC. Tests in absence of photocatalysts were also performed as black reference.

### 5.14 Radical trapping measurements

1 mL of MB aqueous solutions (1 × 10^−5^ M) were mixed with 1 mL of radical- and hole-trapping agent solutions (1 × 10^−3^ M) with absorbance adjusted around 1.0. When the adsorption–desorption equilibrium was reached, all mixed solutions were saturated by bubbling air for 15 minutes for each hour. The cuvettes were exposed to the solar simulation in the same conditions reported above for the photocatalytic test. The MB degradation rate was evaluated by monitoring the absorbance values at 664 nm for two hours. Triplicate data was collected for each irradiation time.

## Author contributions

M. U. and S. C. C. conceived the project and wrote the manuscript. M. U. carried out the SEM images, realized the photocatalytic experiments and analyzed the collected data. M. U., G. C. and D. V. synthesized and characterized the porphyrin copolymer. M. U. and E. B. performed graphene preparation, characterization and developed the etching time-saving procedure. M. Ur. carried out the electrochemical measurements; M. M. made the photoluminescence experiments and G. C. produced the XPS spectra. M. C. carried out the TOC measurements and analyzed the data. S. C. C. and V. P. supervised the project. All the authors reviewed and commented on the manuscript at all stages.

## Conflicts of interest

There are no conflicts to declare.

## Supplementary Material

RA-009-C9RA06328E-s001
