# Supplementary material for: Hybrid nickel-free graphene/porphyrin rings for photodegradation of emerging pollutants in water
Source: RSC Adv. 2019 Sep 24;9(52):30182–94. doi: 10.1039/c9ra06328e (PMC9072085; doi:10.1039/c9ra06328e)
Supplement: RA-009-C9RA06328E-s001 [file RA-009-C9RA06328E-s001.pdf]

Electronic Supplementary Material (ESI) for RSC Advances.
This journal is © The Royal Society of Chemistry 2019

SUPPLEMENTAL INFORMATION

Hybrid Nickel-free graphene polyporphririn photocatalyst: Time-saving material formulation and photo degradation studies

Martina Ussia [1,2], Mario Urso [1,2], Maria Miritello [2], Elena Bruno [1,2], Giusy Curcuruto [3], Daniele Vitalini [3], Guido Condorelli [4], Maria Cantarella [2], Vittorio Privitera [2] and Sabrina C. Carroccio [2,3] *

1 University of Catania, Department of Physics and Astronomy "Ettore Majorana", Via Santa Sofia 64, 95123, Catania, Italy;
2 CNR-IMM Catania Unit, Via Santa Sofia 64, 95123, Catania, Italy;
3 CNR-IPCB, Via Paolo Gaifami 18, 95126, Catania, Italy;
4 University of Catania, Department of Chemistry and INSTM UdR Catania, Viale Andrea Doria 6, 95125 Catania, Italy.

Correspondence to: sabrinacarola.carroccio@cnr.it

Table of content

- **Figure S1(a-b).** MALDI-TOF mass spectra of cyclic porphyrin copolymer;
- **Table S1.** GPC data of cyclic porphyrin copolymer**;**
- **Figure S2.** XPS of Ni-free/G-Porph ring in the Ni2p region;
- **Figure S3.** Cyclic voltammetry of ferrocene;
- **Figure S4.** Cyclic voltammetry of TBAPF6;
- **Figure S5.** Cyclic voltammetry of porphyrin monomer;
- **Figure S6.** UV-Vis spectra of Porph ring;
- **Figure S7.** UV-Vis spectra of 2,4-D before and after the photocatalytic experiment;
- **Figure S8.** Polymer structures assigned to the mass ions appearing in the MALDI-TOF spectra of PEG sample after 6 hours of photoexposure;
- **Figure S9.** MALDI-TOF spectrum of PEG sample after 6 hours of photoexposure at lower mass range;
- **Figure S10.** MB degradation test by adding different concentrations of ABDA;
- **Figure S11.** MB degradation test by adding different concentrations of t-BuOH;
- **Figure S12.** MB degradation test by adding different concentrations of EDTA;
- **Figure S13.** MB degradation test by adding different concentrations of NBT;
- **Figure S14.** Solar simulator apparatus.

MALDI-TOF analysis.

(a)

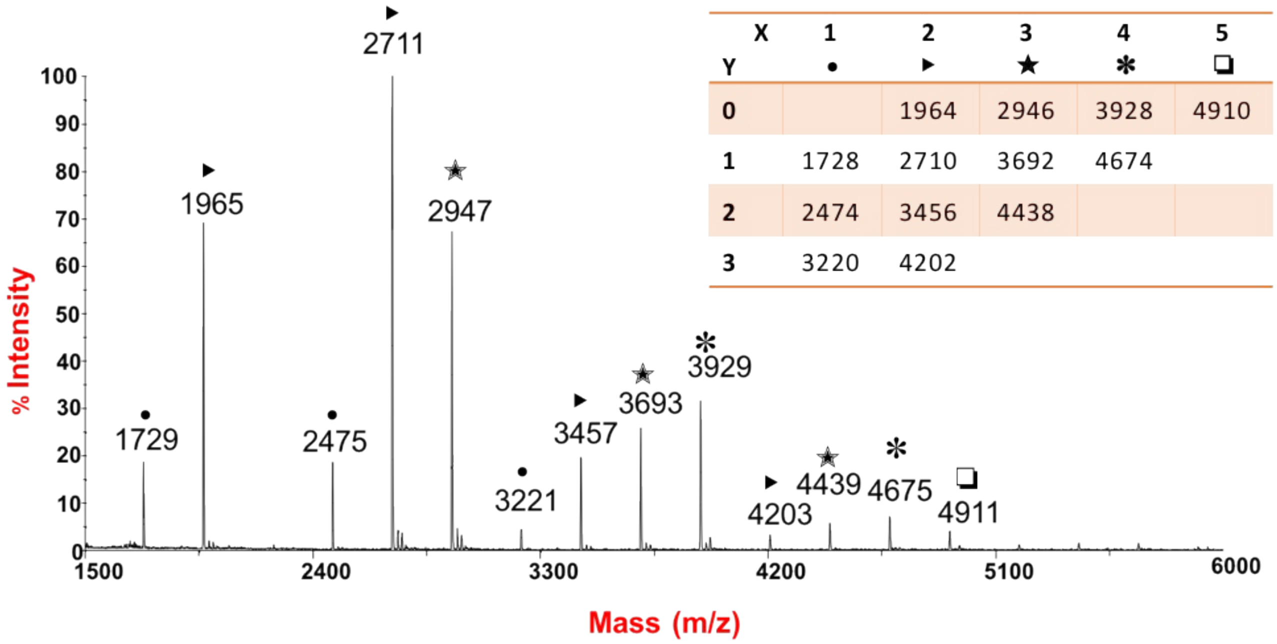

Y \ X	1 •	2 ▸	3 ★	4 ✻	5 ❏
0		1964	2946	3928	4910
1	1728	2710	3692	4674	
2	2474	3456	4438		
3	3220	4202			

(b)

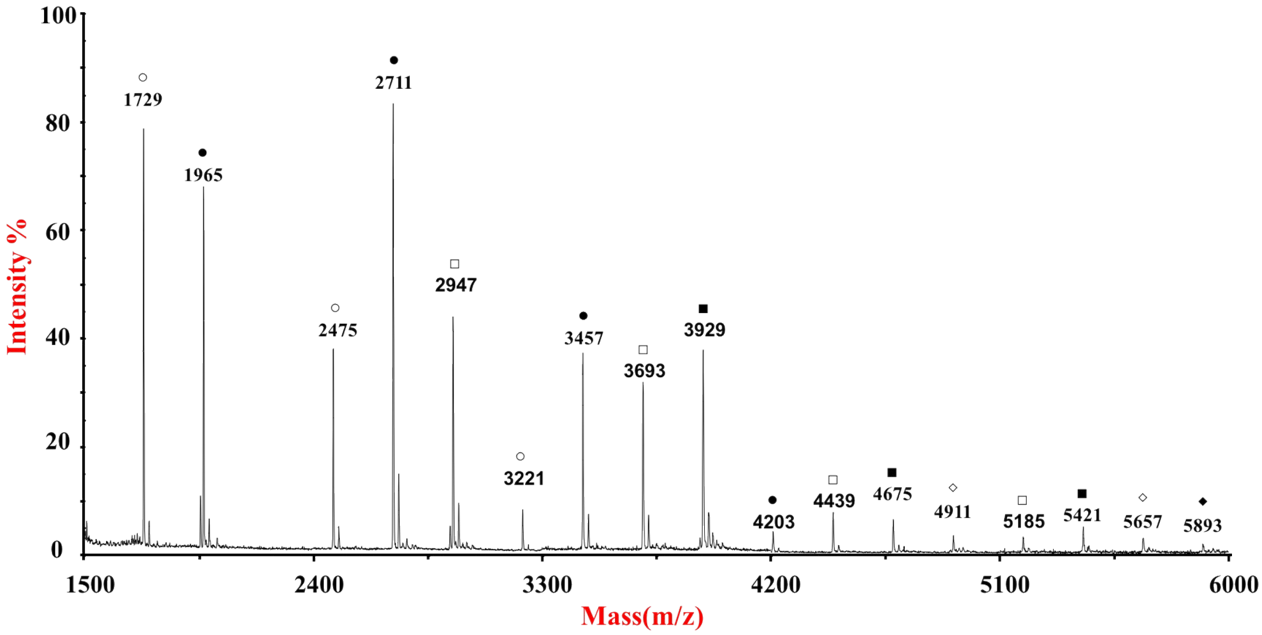

Figure S1. MALDI-TOF mass spectrum, acquired in positive and reflection mode, of **(a)** the porphyrin copolymer with a table for the structural assignments and **(b)** the porphyrin copolymer deposited on Ni Free/G surface.

Table S1.

Composition, yield and Molar Masses of synthesized polymers

Polymer	Nominal % Porphyrin molar content	Actual % Porphyrin molar content	Polymer yield (w/w)[a]	Mw[b]	Mn[b]
**Cyclic copolymer**	50	39%	77%	10580	9300

a) Percent of polymeric material with respect to the total amount of starting monomers
b) Molar masses values calculated by using PMMA as GPC standards.

XPS Analysis.

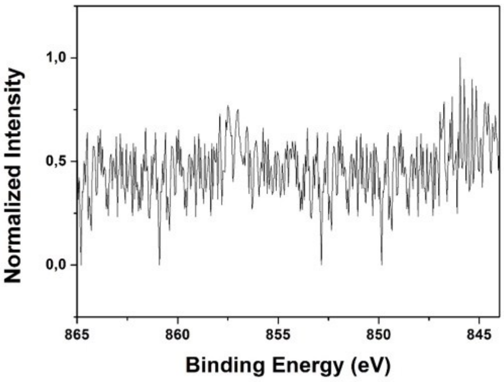

Figure S2. Ni2p region XPS spectrum of Ni-free/3DG Copolymer

Cyclic Voltammetry.

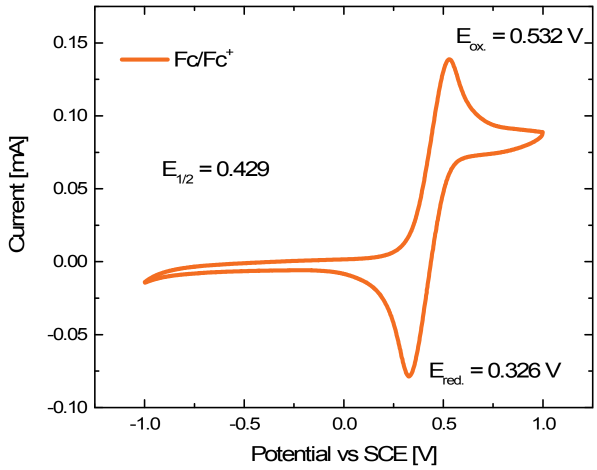

Figure S3. Cyclic voltammetry of ferrocene recorded at 25 mV s^{-1} scan rate in 0.1 M TBAPF6 in dichloromethane solution by using SCE as reference electrode.

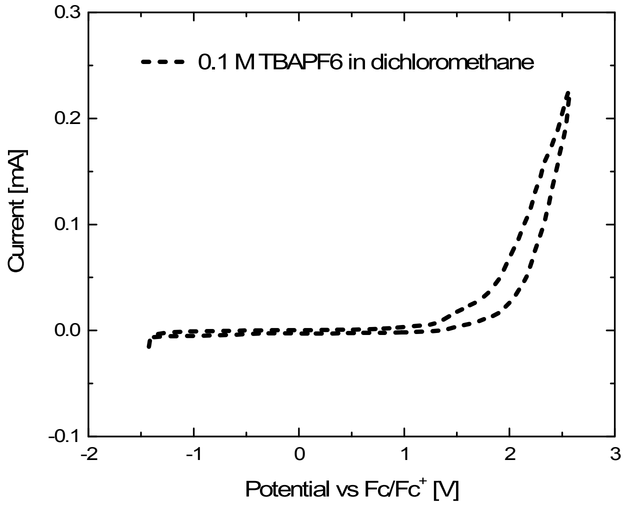

Figure S4. Cyclic voltammetry recorded at 25 mV s-1 scan rate in 0.1 M TBAPF6 in dichloromethane solution. Ferrocene was used as internal standard.

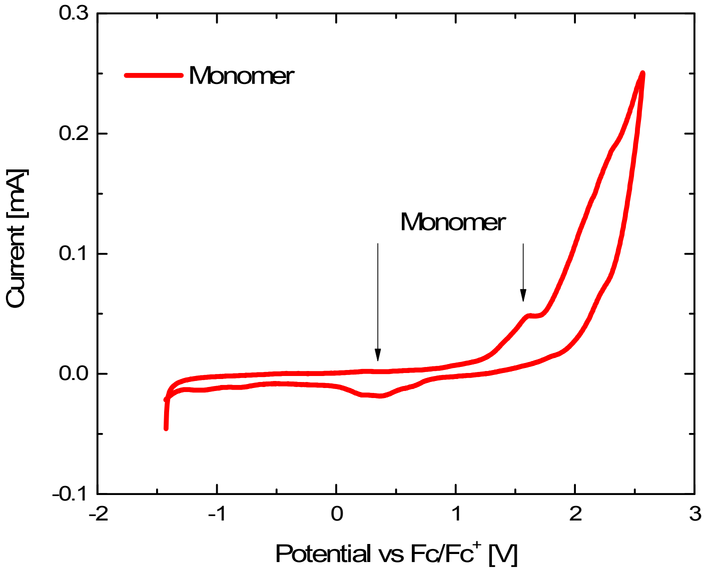

Figure S5. Cyclic voltammetry of monomer recorded at 25 mV s-1 scan rate in 0.1 M TBAPF6 in dichloromethane solution. Ferrocene was used as internal standard.

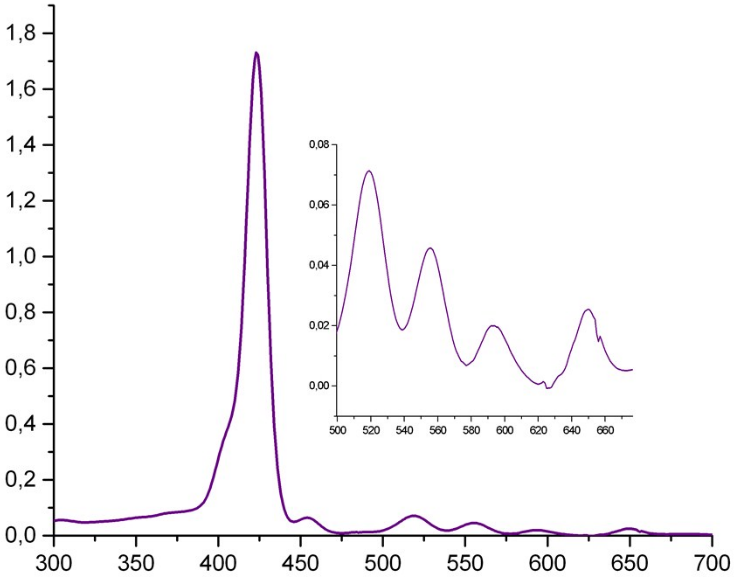

Figure S6. UV-Vis spectrum of porphyrin copolymer in dichloromethane

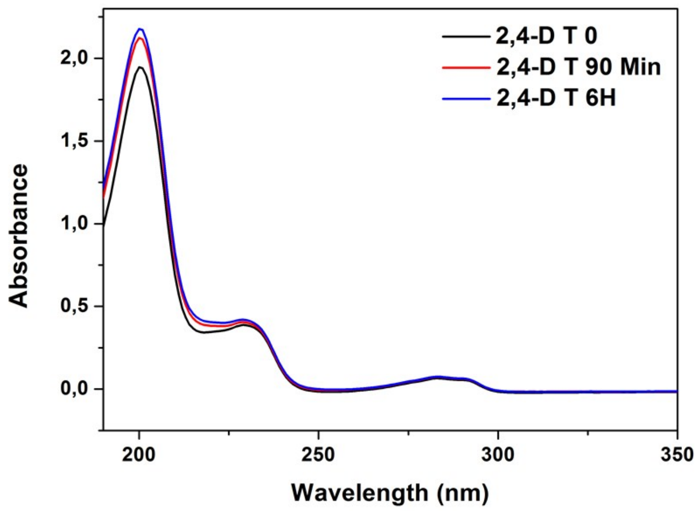

Figure S7. UV-Vis spectra of 2,4-D before and after the photocatalytic experiment.

Structures	MNa^+
$HO-[CH_2-CH_2-O]_8-H$	393.43
$HO-[CH_2-CH_2-O]_8-CHO$	419.45
$HO-[CH_2-CH_2-O]_8-COOH$	437.49
$HO-[CH_2-CH_2-O]_7-CH_2COOH$<br>$HOH_2CO-[CH_2-CH_2-O]_7-CHO$	407.42
$HOH_2CO-[CH_2-CH_2-O]_8-H$<br>$HOH_2CO-[CH_2-CH_2-O]_7-COOH$	423.43

Figure S8. Polymer structures assigned to the mass ions appearing in the MALDI-TOF spectra of PEG sample after 6 hours of photoexposure.

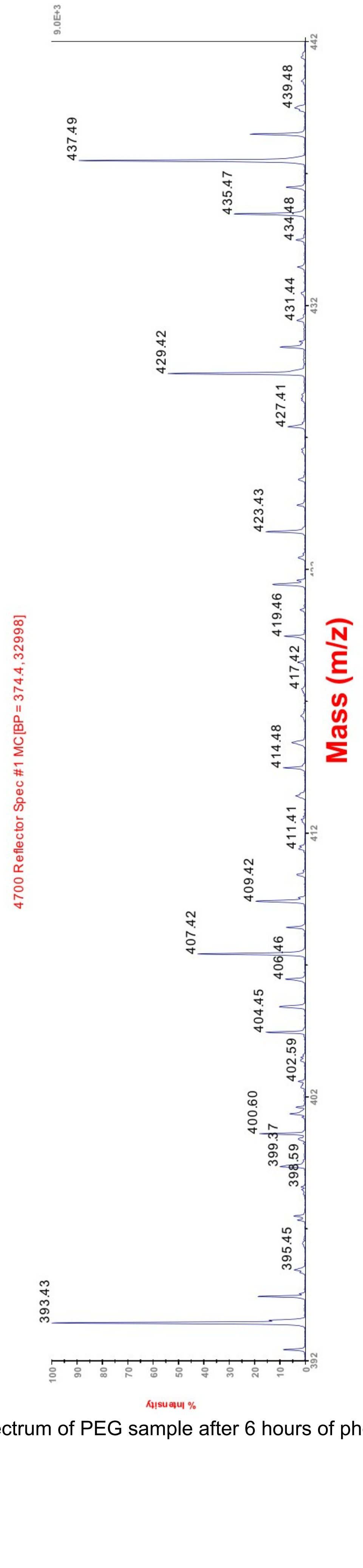

Figure S9. MALDI-TOF spectrum of PEG sample after 6 hours of photoexposure at lower mass range.

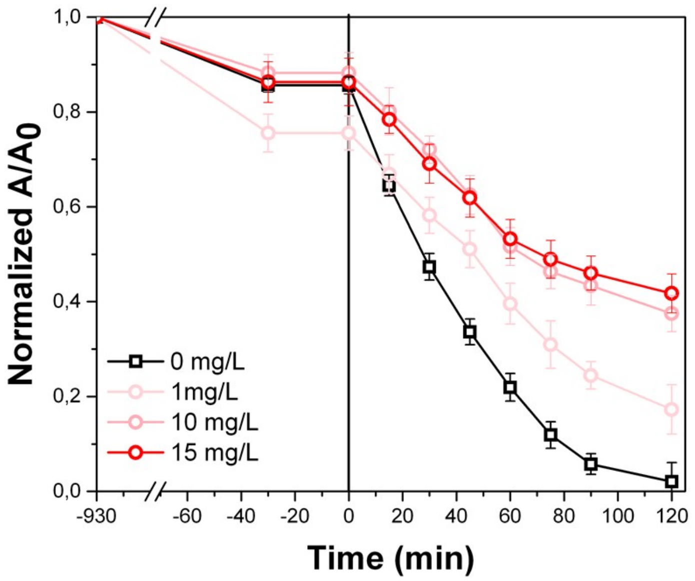

Figure S9. Degradation test of MB after addition of different concentrations of ABDA (0, 1, 10, 15 mg/L) under light irradiation.

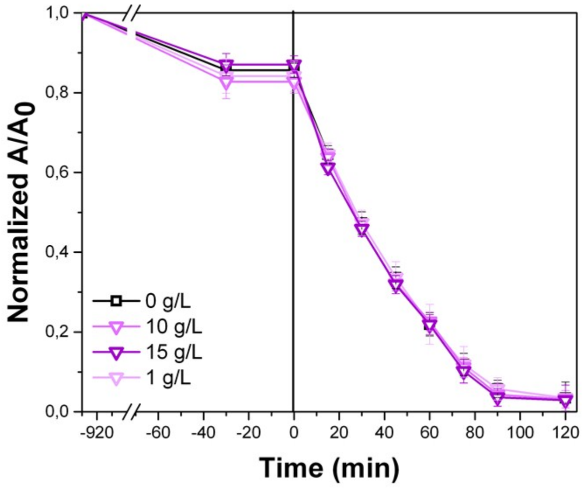

Figure S10. Degradation test of MB after addition of tert-butyl alcohol (0, 1, 10, 15 mg/L) under light irradiation.

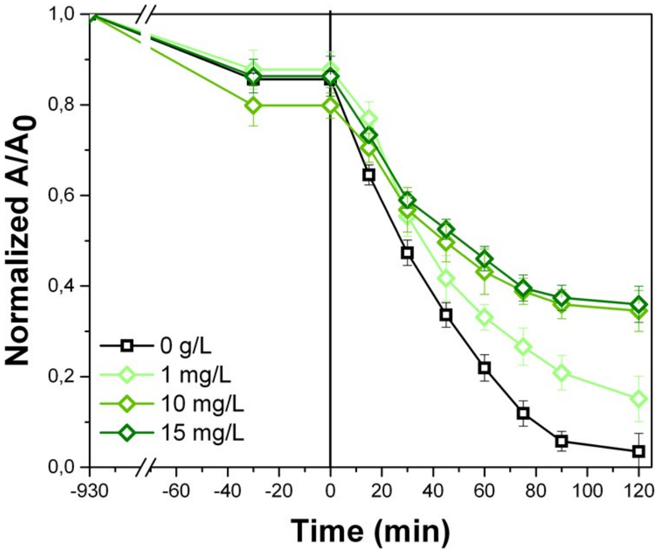

Figure S11. Degradation test of MB after addition of different concentrations of EDTA (0, 1, 10, 15 mg/L) under light irradiation.

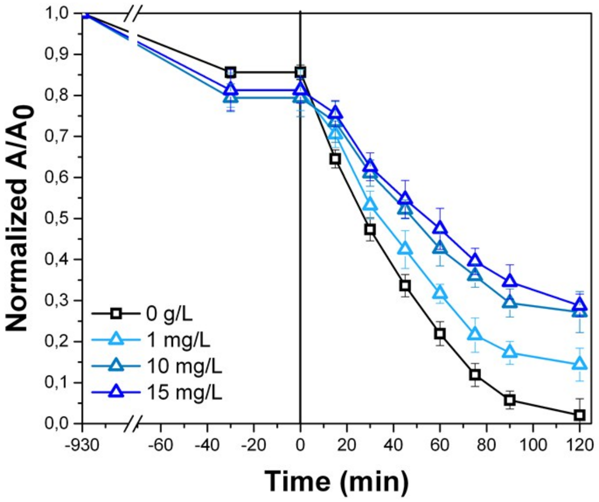

Figure S12. Degradation test of MB after addition of different concentrations of NBT (0, 1, 10, 15 mg/L) under light irradiation.

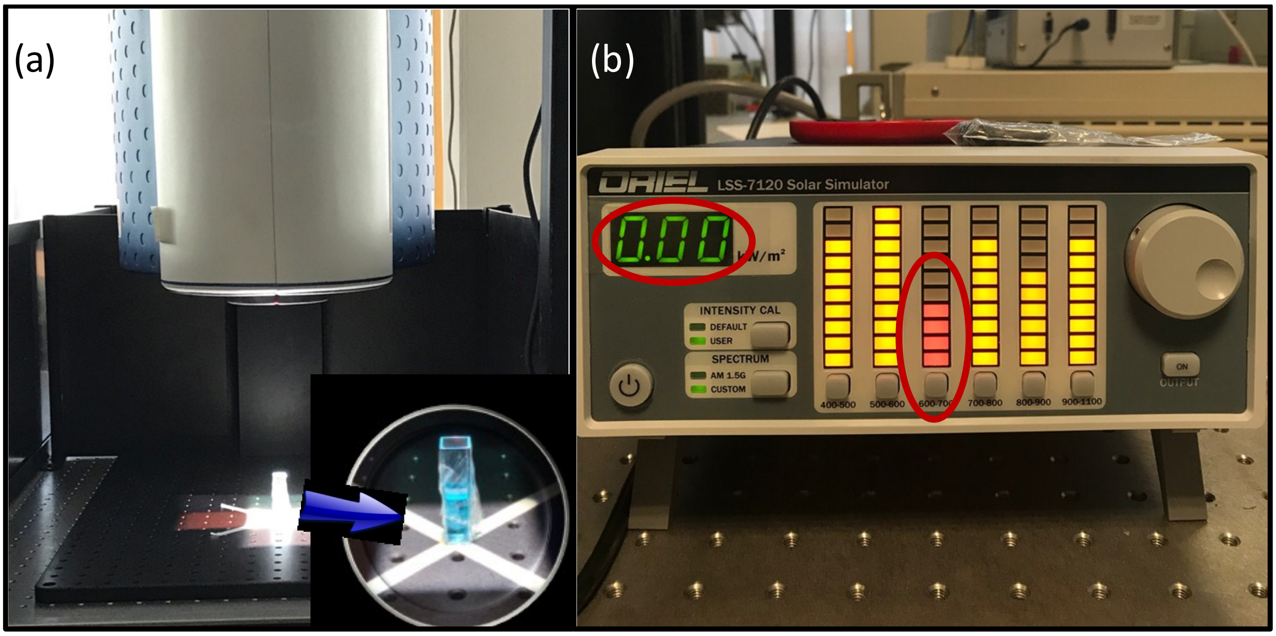

Figure S13. (a) Solar simulator apparatus used to test the photocatalytic performances of the samples and (b) operating conditions used during the photocatalytic tests: irradiation removal in the range of 600-700 nm to suppress the MB absorption.
